# A Process Evaluation of A UK Classroom-Based Physical Activity Intervention—‘Busy Brain Breaks’

**DOI:** 10.3390/children8020063

**Published:** 2021-01-20

**Authors:** Alice Cline, Gareth Knox, Luciana De Martin Silva, Stephen Draper

**Affiliations:** Department of Sport, Hartpury University, Gloucestershire, Gloucester GL19 3BE, UK; Gareth.knox@hartpury.ac.uk (G.K.); Luciana.Silva@hartpury.ac.uk (L.D.M.S.); Stephen.draper@hartpury.ac.uk (S.D.)

**Keywords:** physical activity, fundamental movement skills, classroom based, intervention, process evaluation, RE-AIM framework, primary schools

## Abstract

The gap between development of effective physical activity interventions and the wide-scale adoption of these interventions in real-world settings has been reported since the early 2000s. Evaluations have been criticised for failing to report details of context, implementation, adoption and maintenance. ‘Busy Brain Breaks’ was an intervention designed to improve fundamental movement patterns whilst increasing physical activity within the classroom. This evaluation study used a mixed-methods approach including questionnaires, observations, semi-structured interviews and quantification of class-level dose. Findings suggest that giving teachers flexibility and autonomy over the way in which they implement physical activity interventions may increase the likelihood of adoption. Time was frequently perceived as a significant barrier to the intervention, giving the teachers flexibility to implement the intervention when they thought most suitable allowed teaching staff to retain their autonomy and make the intervention work with their schedule. Children’s behaviour appeared to be both a facilitator and barrier to implementing physical activity interventions within the classroom. Whilst misbehaviour can pose as a barrier, children’s enjoyment acts as a key facilitator to implementation for teaching practitioners. Teachers interviewed (*n* = 17) observed that movement ability had developed as a result of the intervention and recognised co-ordination, balance and stability as areas that had noticeably improved. Conducting an in-depth process evaluation has allowed for greater insight and understanding as to how, and to what extent, the intervention was implemented within the school-based setting.

## 1. Introduction

The World Health Organisation (WHO) identifies schools as primary sites for health interventions due to their ability to reach the vast majority of school-aged youth. On average, school-aged children spend 30 h per week in school, positioning the school environment as a feasible setting for delivering movement and physical activity interventions. In 2019, the UK Ministry of Education suggested that primary schools should be providing their pupils with 30 min of physical activity per day in addition to breaktime and lunchtime [1]. In addition to sedentary teaching practices, opportunities for physical activity outside of the classroom, such as breaktimes and physical education, have decreased as a result of increased focus on academic performance [2]. Therefore, interventions are being designed and implemented to reduce sedentary behaviour and increase physical activity within primary school classrooms.

Naylor and McKay [3] argue that effective physical activity interventions, delivered in settings where children learn, are an important part of the solution. In addition to having the potential to improve multiple health outcomes, there are also many direct benefits to the learner and learning environment such as improved classroom management [4], enhanced cognitive function [5] and improved self-concept [6]. In addition to this, multiple systematic reviews demonstrate the efficacy of school-based approaches [7,8,9]. However, a systematic review conducted by Naylor et al. [10] identified an urgent need for more school-based physical activity studies that assess implementation through comprehensive process evaluation.

The gap between development of effective physical activity interventions and the wide-scale adoption of these interventions in school-based settings has been reported since the early 2000s. Evaluations have been criticised for failing to report details of context, implementation, interventions, adoption and maintenance. Implementation has been defined as a “specific set of activities designed to put into practice an activity or programme of known dimensions” [4,11]. This encompasses all aspects of the process of intervention delivery including the extent to which an intervention and its elements are implemented as planned, how much of the intervention is delivered or received, how responsive participants were to the intervention and changes made to the intervention during implementation that enhance its fit within the setting it is being delivered in [10,12]. It has been argued by Durlak and DuPre [12] that in order to bridge the gap between developed and adopted effective physical activity interventions on a scale broad enough to promote large-scale health changes, there is a critical need to understand factors related to intervention implementation. Understanding these factors within school-based settings is often more challenging due to the notion of schools and the education system itself sitting within a constantly changing broader context [13,14,15]. The RE-AIM framework developed by Glasgow et al. [11] is a health promotion evaluation framework that enables complex settings-based interventions, such as those in school settings, to be comprehensively evaluated. The framework recognises that an intervention may work in theory, but greater consideration is needed as to how factors such as reach, efficacy, adoption, implementation and maintenance affect how the intervention may be received in real-world settings [11]. Using such a framework allows researchers to explore barriers and facilitators to the intervention they are introducing in greater detail.

‘Busy Brain Breaks’ was an intervention designed to improve fundamental movement patterns whilst increasing physical activity inside the classroom for children aged between 7 and 11. Based on the COM-B model and the behaviour change wheel [16]. The behaviour change wheel encourages researchers to think through multiple stages, which encompass various different elements, when designing a behaviour change intervention. Some of these stages include understanding the problem in behavioural terms, selecting and specifying the target behaviour, identifying what needs to change and identifying intervention functions in order to encourage change to happen. At the centre of the behaviour change wheel is the COM-B model, a behaviour system comprising four components that interact with one another—capability, motivation, opportunity and behaviour [16]. Additionally, the intervention was also driven by the experiences and thoughts of current teaching primary school practitioners. This was a result of a previous study in which the researcher conducted focus groups with 12 practitioners from 5 schools across Gloucestershire. The focus groups were conducted in order to explore perceived barriers and facilitators to implementing physical activity inside the classroom, with the discussion and evaluation from the research helping to inform the intervention design.

Aims:

Therefore, the purpose of the current study was to conduct semi-structured interviews which aimed to explore teaching practitioners’ lived experiences of barriers and facilitators when implementing a physical activity intervention in the classroom. Specific aims are below:

(1) Explore teaching practitioner’s adoption and adherence to an intervention aiming to increase physical activity and movement in primary school classrooms across Gloucestershire.
(2) Understand which barriers to implementation were experienced whilst exploring proposed solutions to overcome them.
(3) Discuss maintenance of the intervention in relation to sustained behaviour change.

## 2. Materials and Methods

### 2.1. Participants

Ethical approval was gained by the University’s Ethics Committee (ETHICS 2019-07), and informed consent was gained from the head teacher of each school, the parent and each participating child. In total, 6 schools took part in this study, 3 schools in the intervention group and 3 in the control group. A total of 28 classes and 35 teachers formed the intervention group. As shown in Table 1, 716 (345 boys) students took part in the classroom activity, 553 (240 boys) of these children had consent from their parents to take part in the pre/post intervention testing. Both the intervention and control groups took part in baseline data collection. However, due to COVID-19 causing schools to close, post intervention testing could not take place. Therefore, this paper describes the experiences of the intervention group from the beginning of the intervention to when it was forced to end due to the pandemic.

### 2.2. Procedures

Twenty-eight classes, a total of 716 children, were asked to complete three ‘Busy Brain Breaks’ per day, three times per week at a time that suited the days’ learning activities, for a twenty-week period. The ‘Busy Brain Breaks’ were made up of five minutes of bodyweight exercises following an instructional video. The videos were played on the classroom’s interactive whiteboard and were accompanied by music. As a result of the COVID-19 pandemic, the intervention was cut short at 10 weeks, meaning post intervention data on movement patterns and physical activity levels could not be collected. However, it was possible to conduct a thorough process evaluation using the data collection during the intervention in addition to the data collected from teaching staff in the weeks after the intervention had ended.

At the start of the intervention, each class was provided with a ‘Busy Brain Break’ package. This package included a USB loaded with the videos, an A3 laminated activity tracker chart, progression and regression cards for each exercise and a range of weekly challenges. Teachers and students were asked to keep a record of how many sessions they had completed each week by marking their activity tracker each time they completed a session, and this data was captured by the researcher each Monday morning. During the fifth week, a project-specific questionnaire (please see Appendix A) that focused on the practical elements of the intervention was circulated to each class in order to collect feedback from both teachers and students, a total of 25 questionnaires were returned out of a possible 28. Once the intervention had finished, each teacher was invited to take part in a follow-up phone interview (please see Appendix B), and a total of 17 teachers out of a possible 28 agreed to take part in the data collection. The overall evaluation used a mixed-methods approach including questionnaires, reporting of observations, feedback from teachers and pupils and quantitative dose data. A similar mixed-methods approach was used by Jenkinson et al. [17] and can offer guidance on how to create conditions for successful adoption, implementation and maintenance of future interventions [18].

### 2.3. Data Analysis

The questionnaires and telephone interviews conducted by teachers were analysed using thematic analysis [19]. The interviews ranged from 40 to 55 min in length and were conducted between 9 am and 5 pm, depending on when most convenient for the participant. The interviews were of a semi-structured nature and therefore followed a loose interview guide (please see Appendix A). Given that thematic analysis allows for the identification and organisation of themes within data, it was an appropriate deductive method to use when approaching the data with a framework of pre-existing concepts [20]. All interviews were transcribed verbatim before being read, along with the questionnaires, multiple times in order to promote familiarisation. Once this was completed, the data was coded according to which RE-AIM (reach, efficacy, adoption, implementation and maintenance) framework dimension(s) it was relevant to using nVivo. A total of 15 themes emerged from the data, with some themes having further subthemes. Two authors reviewed these themes to agree on how they worked in relation to the RE-AIM framework, the results of which are presented below. Verbatim quotes have been used by participants, who have confirmed the use of those quotes as they are.

## 3. Results

The definitions of the five components of the RE-AIM framework are frequently adapted by researchers so that they are suitable for the context in which they are applying them to; see Table 2.

### 3.1. Reach

All 10 schools who had taken part in research with the university earlier in the year were invited to take part in the intervention. Six expressed interest and were formally invited to take part, and all 6 accepted and were randomly assigned to either the control group or the intervention group; see Table 3. Two schools that took part in the intervention were large inner-city primary schools, with the third school being smaller in size and semi-rural. All year groups in Key Stage Two (Year 3, Year 4, Year 5 and Year 6) were invited to participate. Prior to baseline testing being conducted, an information letter and consent form were sent home to each parent via the school’s administrator. Once written consent had been obtained from parents, written assent was obtained from the children who wanted to participate in the baseline data collection.

### 3.2. Adoption

#### Teacher’s Adoption

When asked to reflect back on their initial thoughts, queries or concerns about ‘Busy Brain Breaks’ before it started, all 17 teachers spoke about concerns revolving around the practicalities of adopting the intervention. These practicalities involved managing an already busy workload, finding the time to do it and finally being able to make use of the resources. When first introduced, there was apprehension around how much the intervention would impact workload. However, after a week of adopting the intervention, the concerns were no longer an issue.

“I was very aware it was one more thing you know every staff meeting we are concentrating on something different we do have a lot on our plates in terms of workload but once we had started it all my concerns melted away because it didn’t really require anything from me, it wasn’t any extra work to think about”—Participant 2, Teacher of Class 12.

In addition to workload, time was identified as a potential barrier to adoption and implementation of a classroom-based physical activity intervention. This was noted as a frequent concern amongst teachers when first introduced to Busy Brain Breaks.

“First of all I was thinking how are we going to get through 9 in a week, because I know you said it was 3 a day over 3 days and I was thinking oh okay I’m not sure where that’s going to fit in because our curriculum is so tight that was a bit worrying thinking well where is this going to go”—Participant 9, Teacher of Class 9.

Whilst time and workload were identified by teaching staff as the main concerns of adopting Busy Brain Breaks, these factors did not stop them from adopting the intervention. Frequently, teaching staff spoke about having the approval from management as a key factor that helped to overcome these concerns and thus increase likelihood of adoption.

In addition to having the approval from management, barriers to adoption were frequently overcome by the teaching staff’s ability to recognise the benefits of the intervention. This was in part due to the short staff training session delivered by the researcher, but also due to the teaching staff’s knowledge of the benefits of children engaging in more physical throughout the school day.

“I did think oh my goodness me, because it was three times a day, I just thought oh wow that’s three interruptions in the day to do physical activity but I did whole-heartedly believe in the project and I could see the benefit of the project for the children in terms of getting them more active throughout the day”—Participant 12, Teacher of Class 5.

Importantly, teacher confidence was identified as a previous barrier to adoption and implementation of physical activity interventions. Therefore, it was a key objective to make sure the resources were informative, thus giving all teachers confidence to adopt the intervention regardless of prior knowledge of ability. Teachers who had identified themselves as being less confident at delivering physical activity or PE lessons noted that they felt confident adopting Busy Brain Breaks due to the resources and in particular the exercise videos.

“I liked the idea of Busy Brain Breaks despite my confidence because the videos had pretty much everything we needed, so we were able to use the cues, or to pause the video if we needed to all the content was there for us even down to the adjustments to make it easier or harder so in terms of confidence I didn’t ever feel like I wasn’t able to deliver it properly or I didn’t have enough knowledge”—Participant 11, Teacher of Class 15.

In addition to recognising the benefits of adopting the intervention, teaching staff recognised how quickly children adopted Busy Brain Breaks and how much they enjoyed it.

“So, the children were very excited they always love physical activity because they love physical activity really so they were excited to do it”—Participant 12, Teacher of Class 5.

Interestingly, it was suggested that because the children had adopted the intervention so enthusiastically, this increased the teaching staff’s adoption and adherence, with children often reminding their teachers to take a ‘Busy Brain Break’.

Finally, teachers noted that whilst children were quick to adopt Busy Brain Breaks largely due to enjoyment, the intervention also promoted inclusivity amongst the children who may not usually enjoy PE or other physical activities.

“The fact it was just the five minute they know that actually, even the ones who maybe struggle with the physical aspect of it, they can just appreciate that it is just 5 min not that sort of 15 min to run a mile and thought of a mile to some of them sounds quite far whereas this is just five min”—Participant 13, Teacher of Class 17.

This was supported by a number of other teachers who noted that the short bouts of 30 s per exercise were manageable chunks of time for all children, especially those who usually found physical activity and exercise difficult.

“I think it’s a mindset type of thing because they know that it’s short and that’s the really good thing about HIIT style activities is that they are really short and sharp activities so for the children who are finding difficult know that they only have 30 s of each exercise and actually they only have five min of activity in total.”—Participant 9, Class 9.

In addition to the short bouts of exercise, a number of teachers recognised the benefit of having a diverse range of exercises.

“I think the variety of the exercises in Busy Brain Breaks suited all the children for different reasons so some benefited those who had good core strength and others were good at balancing so there was something for everyone and if there was an exercise they weren’t too good at it, well it was only 30 s and then they would move onto something else.”—Participant 11, Class 15.

### 3.3. Implementation

**Figure 1:**
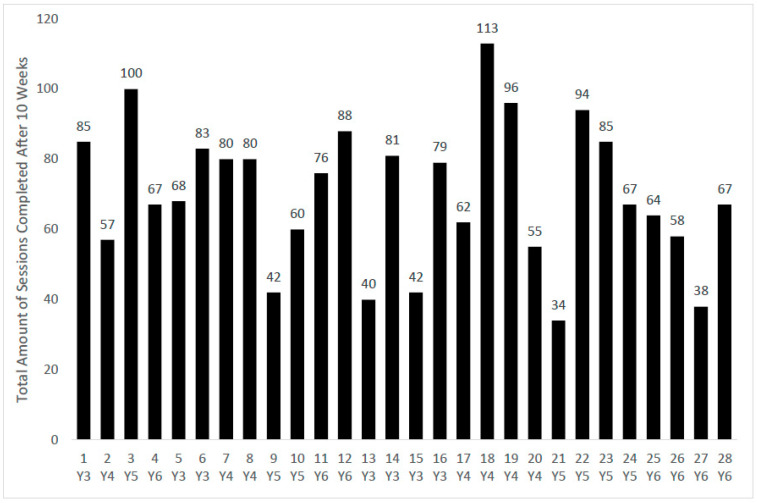
Total number of sessions completed by each class, over the 10 week period.

Out of the 28 classes who took part in the intervention, all 28 teachers adopted the intervention to some extent. The median amount of sessions completed over the 10 week period was 76, with 34 being the minimum amount and 113 being the maximum, as displayed in Figure 1.

When looking at all three schools together, the average amount of ‘Busy Brain Breaks’ completed over the 10 week period was 71.29, meaning, on average, each class completed 7 breaks per week. At the lowest end of the scale, the total amount of ‘Busy Brain Breaks’ completed over the 10 week period was 34, meaning the class was doing an average of 3 breaks per week. At the highest end of the scale, a total amount of ‘Busy Brain Breaks’ completed over the 10 week period was 113, meaning the class was doing an average of 11 breaks per week. Please see Table 4 for a breakdown of how many sessions the 28 classes completed each week.

As noted, a common critique of behaviour change interventions is the lack of understanding as to how the intervention was implemented, and whether or not changes were made during implementation. As a result of the focus groups conducted in study two, having the ability to adapt an intervention aiming to increase levels of physical activity inside the classroom was noted as a key facilitator in order to help teaching staff maintain autonomy. This meant a number of choices regarding implementation were left up to the class teacher.

#### 3.3.1. Time of Implementation

Class teachers were asked to implement 3 busy brain breaks a day, 3 days a week, ideally on non-PE days. It was left up to the teachers to decide exactly when they wanted to implement the three breaks throughout those days. Of the 17 teachers interviewed, 15 of them reported that they regularly implemented a Busy Brain Break between lessons, in what they call the transition period.

“I focused on transitions between subjects so for example in the morning we did guided reading before English and I was finding that to be a struggle because children would talk and drag their heels so we started to a do Busy Brain Break in between and that meant the children would tidy up a lot quicker, instead of them taking 5 min to tidy up they’d be tidying up in 30 s because they’d want to start the video so it was brilliant for the side of things”—Participant 9, Teacher of Class 9.

In addition to encouraging children to finish tidying up from the previous lesson and get ready for the next, teachers also noted that putting a Busy Brain Break in between a lesson helped them to get the classroom ready for the next lesson too.

“I wanted to make it work for me too, I’d do it between lessons so I could prepare for my next lesson whilst walking around the class and giving me feedback. I’m gathering my bits of paper or it might be finding something on the internet that I need whilst watching the class and making sure I’m reading out the cues on the board and what not”—Participant 14, Teacher of Class 19.

In addition to having practical benefits, two teachers commented that it was a time efficient way of implementing the breaks.

“It was really easy to put into action because the 5 min, as soon as they knew what they were doing, we might have had a changeover between two lessons and actually we only lost 2 and a half min out each lesson rather than 5 min out of one so yeah we tried to sandwich it between lessons”—Participant 4, Teacher of Class 3.

#### 3.3.2. Frequency of Implementation

Although teachers were asked to implement Busy Brain Breaks three times a day, three days a week, ideally on non-PE days, it was ultimately left up to them as to how they wanted to structure the nine sessions. Whilst some classes stuck to the proposed structure, the majority of teachers adapted the structure in order to fit in with their weekly schedules.

“So I probably wouldn’t always do the 3, I’d do 2 most of the time and try to do them every day so I’d do 2 one day, then 1 then next day rather than every other day so we were still doing a good amount and that worked with our timetable”—Participant 1, Teacher of Class 26.

Interestingly, 4 teachers noted that implementing Busy Brain Breaks every day, rather than 3 days a week, allowed them to create a routine that was easier to remember and therefore adhere to.

“I just thought right if I try and do 2 a day then worse comes to worse we’ll end up doing 10 so I found that a lot more manageable because you’re not, you’re only having to fit in 2 somewhere, but you’re thinking every day of doing it, whereas if you’re thinking every other day you can easily forget so I actually didn’t end up timetabling them because of that reason.”—Participant 8, Teacher of Class 11.

This was supported further by Participant 6, who suggested it also helped to create a routine for the children too.

“We began by doing it 3 times a day, 3 days a week, but in the end, I definitely found doing it twice a day everyday was better. Because I really liked to have it on set time each day between lessons because then I got used to it and so did the children. So, I forgot the children would remind me.”—Participant 6, Teacher of Class 22.

#### 3.3.3. Changes Made during Implementation

Finally, teachers were asked whether they had to make any changes to the way Busy Brain Breaks was implemented other than time, frequency and the role they played. Out of the 17 teachers interviewed, in addition to the 25 responses via questionnaires, 2 teachers reported making changes to the intervention. Participant 3, teacher of class 24 noted that she implemented an additional minute at the end of each Busy Brain Break where the children practiced mindfulness.

“We used mindfulness or a minute of silence at the end of each session before we got back to work. Sometimes we used GoNoodle, which has great breathing/mindfulness videos just to calm the class back down. Or even just to get the class to sit back in their chairs, close their eyes and think about what they can hear, smell, taste etc. I tried to make sure it didn’t take up more time”—Participant 3, Teacher of Class 24.

Both Participant 3 and Participant 6, teachers of class 24 and class 22, respectively, reported that during the first week of implementation, they used a PE lesson to take their class through a Busy Brain Breaks video.

“I spent a PE lesson in the first week, I spent a whole PE lesson going through it. I went through with them exactly what they needed to be doing. I said to them, I know those of you who are messing around, it’s because you’re finding it hard. And a few of them were nodding, and I said, you’re just being silly because you don’t know how to do it and I get that.”—Participant 6, Teacher of Class 22.

#### 3.3.4. Barriers to Implementation

In order to understand how the intervention was implemented, and in order to help inform future classroom-based interventions, it is important to understand whether there were any factors that acted as barriers to implementation. The most frequently raised barriers were space, time and classroom behaviour. The most frequently reported barrier to implementation was space, with 15 out of 28 class teachers raising this as an issue either through the questionnaires or during interviews.

“Space was definitely a challenge but we just had to make sure we were careful, the children had to look for a space and make sure they weren’t going to bump into anyone as we did the movements and once they were conscious of that it was absolutely fine”—Participant 6, Teacher of Class 22.

In addition to space, time was also reported by 11 teachers as being a barrier to implementation. Given that time was raised as a potential barrier to adoption and implementation during the focus groups conducted in study two, this was to be expected. Time was most frequently mentioned by the Year 6 class teachers, caused by the busy work schedule as a result of SATs.

“Time especially I don’t know if it’s different in year 6 I’m not sure but because we’ve got so much to fit in in such a short space of time, obviously now we haven’t because we have got SATS, but if we had SATs you’ve got to fit it all in by May so that makes it quite a struggle to fit in all 3 which is why I didn’t get to do more Busy Brain Breaks a day”—Participant 1, Teacher of Class 26.

The final challenged, noted six classroom teachers out of the possible 28, was classroom behaviour. Of the six teachers who identified behaviour as a barrier to implementation, five noted that it only lasted as a barrier for the first two weeks of the intervention. All six teachers noted the importance of identifying and addressing the misbehaviour during Busy Brain Breaks early on in the intervention in order to prevent it from affecting further implementation.

“I mean every child is different, the way they react, but yeah nipping it in the bud early was important for me and for them because then they knew what was acceptable and what wasn’t”—Participant 7, Teacher of Class 1.

#### 3.3.5. Facilitators to Implementation

Whilst misbehaviour posed a challenge to implementation at the start of the intervention, eight teachers identified being able to use the way they implemented Busy Brain Breaks to help with classroom management. For some teachers, the threat of taking Busy Brain Breaks away helped them to manage classroom behaviour. Others found offering Busy Brain Breaks as reward for completing or finishing a task quickly was beneficial.

“I’d say okay you’ve got twenty min left and we’re going to work for 15 min of that but if you finish your work because they can be talkative in the afternoon especially so if you finish your work in the next 15 min then great we can do a Busy Brain Break but if not then we won’t be able to do it so yeah from a behaviour management point of view as well it’s like that carrot at the end of the stick and it is a big carrot because they really enjoy Busy Brain Breaks”—Participant 13, Teacher of Class 17.

Good-quality resources were identified as a potential facilitator to both adopting and implementing a classroom-based physical activity intervention during the focus groups conducted during study two. Twenty-two of the 28 class teachers identified the resources as positive influence throughout implementation. Importantly, the instructional cues and practical demonstration of the exercise within the videos allowed teachers to confidently instruct children despite their own physical activity knowledge and ability.

“Even if you don’t have the knowledge you’ve still got the videos on the screen and you know even if you didn’t know exactly why they had to be in that position you can still show a child the video and even demonstrate it yourself to help get them into that position”—Participant 8, Teacher of Class 11.

### 3.4. Efficacy

For this study, efficacy considers how the intervention influenced primary outcome changes, as well as assessing whether positive or negative outcomes were experienced by individuals or within the school setting. This was measured through questionnaires and follow-up interviewers with both teaching staff and pupils. It was intended for this outcome to be measured using the Athlete Introductory Movement Screen (AIMS-4). However, due to the COVID-19 pandemic, it was not possible to collect the post intervention measurements. The successors identified fell into either physical successors such as movement ability, or behavioural-educational successors such as time-on-task behaviour. It can therefore be argued that the behavioural-educational successors are likely to be higher in validity in comparison to the physical successors as the participants were all primary school practitioners.

#### 3.4.1. Movement Ability

All 17 of the teachers interviewed identified that movement ability had improved as a result of the intervention. The teacher’s previous knowledge and experience affected how in depth they were able to discuss these improvements, but despite previous knowledge, all reported observing a noticeable change in the way in which children were performing the exercises. The majority of teachers were able to identify specific exercises that the children improved in. The most frequently mentioned were press ups, deadbugs and planks.

“In terms of my opinion of the results, the results are obvious you know when we started maybe two of them could do some half-hearted press-ups whereas more recently the majority of them are doing proper press-ups during the press up bit, or planks were spent with their backsides in air or even lying down by the end lots of them were doing planks for the duration and they actually looked like the ones being done on the video. The deadbugs were all over the place to start but then they obviously improved their co-ordination a lot and weren’t getting mixed up or confused anymore.”—Participant 8, Teacher of Class 11.

#### 3.4.2. Fitness and Physical Activity

Seven teachers identified a positive improvement to the children’s fitness levels whilst they were completing Busy Brain Breaks.

“I know definitely at the start they were moaning and they found it hard and they were out of breath and I even had a couple of parents say their kids had aching legs the next day but then over time it definitely got easier and they wouldn’t moan and they could do the whole 5 min without needing a rest and they even commented to me about how they were finding it easier too and they weren’t having to take as many breaks”—Participant 6, Teacher of Class 22.

In addition to movement ability and fitness levels, eight teachers during interview and three teachers via questionnaire identified the effect that the intervention had on general physical activity levels.

“Definitely and the effect of Busy Brain Break wasn’t just specific to when we were doing the actual exercises because the children would talk about it before and after and they’d often go out to break talking about various exercises, I had a small group of children who would come in with different variations on exercises that we had done so I think we had a few different types of lunges and they found different ways to do planks so it helped to encourage more chat about fitness and exercise which was nice”—Participant 9, Teacher of Class 9.

#### 3.4.3. Time-on-Task Behaviour

In addition to physical outcomes, all seventeen teachers noted positive behavioural and educational benefits as a result of the intervention. The most frequently identified benefit was improved focus, which had a positive impact on time-on-task behaviour.

“Oh, it definitely re-focuses them, it kind of draws a line, you draw a line under the activity that they have just done and they take a big breath and they are ready, they are just more ready to learn.”—Participant 14, Teacher of Class 19.

All 17 teachers recognised that children were required to sustain concentration for long periods of time throughout the school day. It was suggested that being able to stand up and release some energy was the mechanism behind the improved focus and improved time-on-task behaviour.

“They’re expected to sustain concentration for long periods of time and you know that’s difficult and they can maybe do it for 30 min if you’re lucky and then they start getting chatty and you can hear the noise level in the room change so being able to give them a chance to get up and get moving really helps them to get rid of that energy and they are able to sit back down and concentrate on their work.”—Participant 7, Teacher of Class 1.

### 3.5. Maintenance

When asked whether they would implement the intervention again next year, sixteen out of the seventeen teachers interviewed reported that they would use Busy Brain Breaks in their classroom again next year. Out of the sixteen teachers who reported that they would deliver the intervention again next year, three teachers noted that they would make some changes to the intervention. The most frequently mentioned change was the frequency of Busy Brain Breaks, with the teachers suggesting they would do fewer breaks each week due to time constraints.

“I think probably for me to make it more manageable I’d have to do say like 2 maybe 3 times a week so 6 a week which I think for me is more manageable.”—Participant 1, Teacher of Class 26.

As a result of the COVID-19 pandemic, the Busy Brain Break videos were uploaded to YouTube and each teacher was asked to make their classes aware that they could access the videos at home. As part of the questionnaire sent home to children via their school website, children were asked whether they were doing Busy Brain Breaks at home during the pandemic.

The results indicate that 75.75% of children who completed the questionnaire were doing Busy Brain Breaks at least once a week at home. When separated by school, the average amount of sessions being completed at home differs, with children from school A having a median value of 5 sessions per week, children from school B having a median value of 3 sessions per week and children from school C having a median value of 1 session per week.

## 4. Discussion

By using the RE-AIM framework to conduct a process evaluation, this paper has been able to draw out key barriers and facilitators to a small-scale physical activity intervention implemented across 28 classrooms in Gloucestershire, UK. Three schools, two of which were large inner-city primary schools, were randomly allocated to the intervention group, with the third school being smaller and semi-rural. It is worth noting that the three schools who took part in the intervention may have been more likely to take part due to their willingness to engage in physical activity. Consideration therefore needs to be given to school settings who may be less willing, or less likely, to engage in interventions aiming to increase physical activity levels.

Upon reflection, teaching practitioners made it clear that, prior to implementation, they had concerns revolving around the practicalities of adopting a new intervention. These included managing an already busy workload, finding the time to implement the regular movement breaks as well as being able to make use of the resources. Struggling with finding the time to implement physically active breaks inside the classroom has been identified as a barrier by previous research [21,22,23]. It could be argued that English primary schools have seen an increase in cultures of performativity over the past decade. Troman et al. [24] identifies target setting, Ofsted inspections, school league tables, performance management and performance-related pay as systems that demand teachers to ‘perform’ and to be individually accountable. Whilst these measures have been introduced to improve students’ achievements, they often have a negative impact on teacher’s workload, their professional identities and their experience of teaching [24,25]. This is supported by previous research which identifies pressure to perform well in assessments as a barrier to increasing physical activity throughout the school day [26].

The findings of this evaluation suggest that giving teachers flexibility and autonomy over the way in which they implement an intervention may increase the likelihood of adoption. Introducing an intervention with high-quality resources that are engaging for both teachers and children also acts as a key facilitator to adoption. Ease of adoption is an important factor for any behaviour change intervention to consider. Given the identified time constraints, it is logical that teachers would prefer resources that require little effort on their behalf. A systematic review conducted by Naylor et al. [3] identified quality of resources as the most important facilitator to physical activity intervention implementation. Being able to adapt an intervention or producing an intervention that is flexible in its approaches created an interesting topic for consideration. On the one hand, guidelines suggest that interventions should be delivered with precise consistency to all of their participants, with studies failing to do so facing critique [27]. On the other hand, it is often the case that ‘one size’ does not fit all, and the adoption of an intervention often needs to be adapted to fit the participants’ needs [28].

As a result of the present study, the intervention was successfully implemented to some extent by all 28 teachers. Interestingly, a recent review conducted by Calvert et al. [29] notes that consideration of factors such as organisational climate directed towards teaching practitioners is critical to school-wide implementation of behaviour change interventions. The use of the COM-B model and the behaviour change wheel encourages careful consideration of multiple factors—in this case, both the children’s and teacher’s capabilities, opportunities and motivations. This may help to explain why the intervention was well received. Time was frequently perceived as a significant barrier to the intervention, and giving the teachers flexibility to implement the 5 min videos when they thought most suitable allowed teaching staff to retain their autonomy and make the intervention work with their schedule. Research suggests that an individual is more likely to want to engage in behaviour change if they feel as though it is their choice to do so, rather than having the choice made for them [30]. This degree of perceived autonomy refers to an individual’s willingness to engage in behaviour change and can therefore heavily influence motivation [31]. Children’s behaviour appears to be both a facilitator and barrier to implementing physical activity interventions within the classroom. Whilst misbehaviour can pose as a barrier, children’s enjoyment acts as a key facilitator for teaching practitioners. This concern is supported in the wider literature that has previously identified pupil behaviour as a barrier to implementation [21,23] Quarmby et al. [23] note that ensuring pupils remain seated throughout a lesson helps to ensure a level of classroom control and management, which teachers could be hesitant to disrupt. Future research should therefore focus on support for teaching practitioners to help manage potential misbehaviour during physical activity interventions, in order to prevent it from becoming a barrier.

All 17 of the teachers interviewed identified that movement ability had improved as a result of the intervention ‘Busy Brain Breaks’, with teachers recognising co-ordination, balance and stability as areas that had improved the most. In addition to movement, seven teachers identified that fitness levels had improved during the intervention, with children having to take fewer and shorter rest breaks during the exercises. This is potentially significant considering low cardiorespiratory and muscular fitness have previously been associated with reduced metabolic health in children and adolescents [32,33]. It must be noted, however, that cardiorespiratory and muscular fitness were not assessed directly in the present study and that objective assessment would be required to substantiate the teachers’ observations. Considering motor competence has previously been linked to cardiorespiratory and muscular fitness [34], it is possible that the intervention had a positive effect on these health-related factors. Some teachers noted that physical activity outside of the intervention had also been positively impacted, with children talking more frequently about physical activity and practicing the exercises at home. This is perhaps a reflection of increased enjoyment of physical activity, which has recently shown to be an important predictor variable for achieving physical activity guidelines in primary school children [35]. Authors suggested that enjoyment of physical activity should be an important aspect when designing future interventions, and although enjoyment was not assessed directly in the present study, comments from teachers would appear to support this recommendation.

A limitation of this study is the lack of objective pre/post intervention measurements. It was intended for these outcomes to be measured using the Athlete Introductory Movement Screen (AIMS-4). These objective measurements were to be presented alongside the process evaluation data to help support the interventions effectiveness. However, due to the COVID-19 pandemic, it was not possible to collect the post intervention measurements due to schools being forced to close. In order to collect efficacy data, teachers were asked about their perceptions of general successors as a result of the intervention. It is important to note that findings for efficacy are considerably less valid than they would be if objective measures had been taken.

All 17 teachers interviewed noted that the intervention improved focus, which positively impacted time-on-task behaviour. Teachers also noted that the intervention had a positive impact on peer work, with children frequently giving each other feedback and encouragement. These findings are similar to that of Donnelly and Lambourne [6], who found a link between physical activity, cognitive function and academic achievement. More recently, a systematic review conducted by Daly-Smith et al. [27] identified classroom movement breaks as being successful methods for increasing overall time spent doing physical activity during the school day and for improving classroom behaviour. It has been suggested that giving children a break from concentration and a chance to release their energy is a key mechanism for improved focus [22]. Furthermore, research suggests that making this break an active one has both physical and educational benefits, as identified by Norris et al. [36].

Whilst this study was planned to last for 20 weeks in order to understand how the intervention was implemented over a longer period of the time, the pandemic meant that the intervention lasted for 10 weeks. As Barnett et al. [37] note, whilst physical activity interventions that aim to increase levels of physical activity and improve movement skill can be effective, less is known about the longitudinal results. Currently, this study has only reported the teacher’s intention to maintain their behaviour change. Therefore, follow-up research in 12 months’ time would be advantageous to further understand whether the intervention is able to create sustained behaviour change.

Finally, it is worth noting that children and teachers were able to carry on delivering and using ‘Busy Brain Break’ videos when the pandemic closed schools in April. Given the uncertain nature of the current pandemic era, future research may benefit from exploring the use of digitally delivered physical activity in further detail. Technology has the potential to allow children to maintain physical activity levels at home, or to engage in physical activity at school whilst being able to maintain a social distance from their peers.

## 5. Practical Implications

In addition to conducting a thorough process evaluation using the RE-AIM framework, it was important to collect what the teachers perceived to be important guidance for successful delivery of the intervention. At the end of each interview, teachers were asked to reflect back on their experience of Busy Brain Breaks and give three top tips for someone who was hoping to successfully implement the intervention inside their classroom in the future. The advice has been summarised below.

- Introduce the children to Busy Brain Breaks by showing them a video first and talking through the benefits of physical activity.
- Set out clear behavioural expectations before implementing Busy Brain Breaks inside the classroom.
- Make sure the children find a space and are aware of who is behind them, to the side and in front of them.
- Ask for quiet voices, or little to no talking when the videos are on in order to help reduce silly behaviour.
- Try to do some of the exercises with the children where possible.
- Be sure to give lots of positive reinforcement to all children, especially the children who find physical activity difficult or do not enjoy it.
- Have a rough idea in your head as to when you plan to implement the Busy Brain Breaks throughout the day.
- Do your best to stick to the schedule in your head and finish/start lessons promptly so you are being time efficient.
- Try to keep consistent with the days and times so that it becomes part of your class’s everyday routine.

## Figures and Tables

**Table 1:** Number of pupils with consent per year group.

	School	Year 3		Year 4		Year 5		Year 6	
		No. of Pupils in Year Group	No. of Pupils with Consent	No. of Pupils in Year Group	No. of Pupils with Consent	No. of Pupils in Year Group	No. of Pupils with Consent	No. of Pupils in Year Group	No. of Pupils with Consent
Intervention	A	29	23	29	23	29	25	19	15
	B	60	42	60	46	60	39	59	39
	C	120	76	121	68	120	91	120	66
Control	D	30	15	20	12	30	15	29	6
	E	29	18	27	13	28	20	29	23
	F	51	2	58	11	52	38	38	21

**Table 2:** RE-AIM (reach, efficacy, adoption, implementation and maintenance) health promotion evaluation framework terminology relevant to the Busy Brain Breaks intervention at both the individual and settings levels, adapted from the definitions provided by Jenkinson [11].

Term	Definition/Measurement
**Reach**	Refers to the representativeness of the school and the individual’s willingness to participate in the study. Reasons for non-participation were included after being gathered from teachers and participating leaders. Measured using nationally available school data in addition to questionnaire data.
**Efficacy**	Considers the effectiveness of the intervention at influencing primary outcome changes, as well as assessing whether positive or negative outcomes were experienced by individuals or within the school setting. This was measured through questionnaires and follow-up interviewers with both teaching staff and pupils.
**Adoption**	Refers to the school’s acceptance of the intervention within the organisation and examination of factors that influenced that decision. This was measured through questionnaires.
**Implementation**	Refers to the extent to which the participating students and school completed and made use of the various components of the intervention including barriers and facilitators to implementation. This was measured by the level to which the main components, activities and evaluations were completed as intended.
**Maintenance**	Refers to the extent to which schools and leaders maintained, continued or planned to continue with the intervention. This was measured through follow-up phone interviews with teaching staff.

**Table 3:** Descriptive data for participating schools.

Variable	School A	School B	School C	School D	School E	School F
**Location**	Rural	Inner City	Inner City	Inner City	Semi-Rural	Semi-Rural
**Deprivation Indices**	29,997	32,390	30,776	13,914	23,747	22,762
	10% least deprived NBH in the country	10% least deprived NBH in the country	10% least deprived NBH in the country	50% most deprived NBH in the country	30% least deprived NBH in the country	40% least deprived NBH in the country
**Type of School**	Maintained	Maintained	Maintained	Maintained	Maintained	Maintained
**Number of Pupils**	173	416	482	195	265	393
**% Pupils Eligible for FSM**	7.5%	7.9%	6%	16.4%	6%	15.8%
**Ofsted Rating**	Good	Good	Good	Good	Good	Good

FSM = free school meals; NBH = neighbourhoods.

**Table 4:** Total amount of Busy Brain Breaks completed by each class over 10 week period.

School	Class	Year	Week/Dose										
			1	2	3	4	5	6	7	8	9	10	TotalDose
**A**	1	Y3	8	9	8	9	7	5	9	11	10	9	85
	2	Y4	10	6	0	7	6	4	4	6	7	7	57
	3	Y5	9	9	9	10	10	9	10	11	12	11	100
	4	Y6	6	9	6	2	6	4	10	7	8	9	67
**B**	5	Y3	7	9	6	9	7	5	4	5	7	9	68
	6	Y3	8	7	8	6	9	9	9	9	9	9	83
	7	Y4	8	7	8	8	8	8	8	8	9	8	80
	8	Y4	11	8	10	11	8	3	6	7	8	8	80
	9	Y5	4	10	5	2	3	0	4	4	5	5	42
	10	Y5	7	7	9	7	3	5	5	6	5	6	60
	11	Y6	6	9	6	9	7	9	8	5	8	9	76
	12	Y6	9	10	11	10	8	9	9	6	8	8	88
**C**	13	Y3	3	5	4	4	4	4	4	4	4	4	40
	14	Y3	4	4	7	8	11	9	9	10	9	10	81
	15	Y3	5	5	4	4	4	4	4	3	5	4	42
	16	Y3	8	6	7	8	8	7	9	9	9	8	79
	17	Y4	5	9	7	7	6	5	6	5	6	6	62
	18	Y4	13	13	11	12	13	9	11	10	11	10	113
	19	Y4	12	9	13	10	10	8	8	9	9	8	96
	20	Y4	9	8	5	5	7	3	4	5	5	4	55
	21	Y5	10	9	8	1	1	1	1	1	1	1	34
	22	Y5	11	11	10	10	5	10	8	9	10	10	94
	23	Y5	9	9	9	9	9	8	7	8	8	9	85
	24	Y5	0	6	3	5	10	8	7	9	10	9	67
	25	Y6	3	7	7	6	6	7	7	8	7	6	64
	26	Y6	5	4	6	6	5	4	8	6	7	7	58
	27	Y6	7	4	6	3	2	3	3	4	3	3	38
	28	Y6	7	2	7	7	7	6	8	7	8	8	67

## Data Availability

The data presented in this study are available on request from the corresponding author. The data are not publicly available due to the data containing information that could compromise the privacy of research participants.

## Appendix A

**Process Evaluation Questionnaire (to be completed during the intervention)**
School:
Teacher:
Year Group:
Class Name:
(1) How are Busy Brain Breaks going?
(2) How are you implementing Busy Brain Breaks?
(3) Are you implementing them as originally planned?
(4) Have you had to change that way you implement them at all?
(5) Have you noticed any consequences (positive or negative) of Busy Brain Breaks?
(6) Is there anything stopping you from doing Busy Brain Breaks more regularly?
(7) Do you have any questions/concerns regarding Busy Brain Breaks?

## Appendix B

Follow-Up Semi-Structured Interview Schedule
**Teaching Background**
Can you tell me a bit about your teaching background to begin with?
*How long have you been teaching?*
*How long have you been at your current school?*
*How long have you been teaching this year group?*
*Do you have a specialist subject or additional responsibility?*
What has your experience of delivering PE and/or physical activity been?
*Do you deliver your own PE lessons?*
*Have you delivered much physical activity outside of PE?*
*What are your thoughts on delivering physical activity outside of PE?*
How did you feel about Busy Brain Breaks before they started?
*Thoughts?*
*Queries?*
*Concerns?*
**Initial Implementation: What happened at the start?**
What happened when Busy Brain Breaks was first introduced inside the classroom?
*Can you remember what you were thinking?*
*Can you remember the children’s reactions/thoughts?*
*Can you give me an example?*
Can you tell me about the initial impact it had on the class at the beginning?
*Positive impact?*
*Negative impact? If so, what did you do?*
*Can you give me an example?*
Did you have to make any changes to the way in which Busy Brain Breaks were implemented?
*Can you give me an example?*
**Overall Successors: What was successful over the 10 weeks?**
In what ways has Busy Brain Breaks been successful in your class?
*Positive changes?*
*Movement?*
*Physical activity?*
*Time on task?*
Can you tell me about what the children thought was successful/good?
*Can you give me an example?*
**Overall Challenges: What was challenging over the 10 weeks?**
Did you face any challenges as a result of Busy Brain Breaks?
*Challenges in delivery?*
*Classroom behaviour?*
*Time?*
*Can you give me an example?*
*Did anything stop you from overcoming these challenges?*
What suggestions would you give someone for overcoming those challenges?
*What could be done differently?*
*Why did you do it that way?*
*Can you give me an example?*
Previous focus groups identified possible barriers such as time, space, a busy workload, teacher confidence, resources, fear of getting it wrong, classroom behaviour
*What is your experience of these challenges?*
*Can you give me an example?*
Can you tell me about what the children thought was challenging/hard?
*How did you overcome this?*
*Can you give me an example?*
**Sustainability:**
Would you consider delivering Busy Brain Breaks again next year?
*If yes, why?*
*If no, why?*
Would you make any changes to Busy Brain Breaks?
*If so why?*
*Can you give me an example?*
Reflecting on your experience, if you were to advise someone who wanted to implement more physical activity inside the classroom, what would be your top 3 tips?
*Do’s or dont’s?*
